# Associations of Health Club Membership with Physical Activity and Cardiovascular Health

**DOI:** 10.1371/journal.pone.0170471

**Published:** 2017-01-20

**Authors:** Elizabeth C. Schroeder, Gregory J. Welk, Warren D. Franke, Duck-chul Lee

**Affiliations:** 1 Department of Kinesiology and Nutrition, University of Illinois at Chicago, Chicago, Illinois, United States of America; 2 Department of Kinesiology, Iowa State University, Ames, Iowa, United States of America; Texas A&M University, UNITED STATES

## Abstract

**Introduction:**

This study evaluates whether a health club membership is associated with meeting the US physical activity (PA) guidelines and/or favorable cardiovascular health.

**Methods:**

Using cross-sectional data of health club members (n = 204) and non-members (n = 201) from April to August 2013, this is the first study to our knowledge to examine a health club membership in relation to objectively measured cardiovascular health indicators including resting blood pressure, resting heart rate, body mass index, waist circumference, and cardiorespiratory fitness based on a non-exercise test algorithm. To determine the total PA and sedentary time, this study used a comprehensive PA questionnaire about both aerobic and resistance activities at the health club, as well as lifestyle activities in other settings, which was developed based on the International Physical Activity Questionnaire (IPAQ).

**Results:**

The odds ratios (95% confidence interval) of meeting either the aerobic, resistance, or both aerobic and resistance PA guidelines for members compared to non-members were 16.5 (9.8–27.6), 10.1 (6.2–16.3), and 13.8 (8.5–22.4), respectively. Significant associations of health club membership with more favorable cardiovascular health outcomes and sedentary behavior were observed for resting heart rate (B: -4.8 b/min, p<0.001), cardiorespiratory fitness (B: 2.1 ml/kg/min, p<0.001), and sedentary time (B: -1.4 hours, p<0.001). Participants with a health club membership of >1 year had more favorable health outcomes, with a smaller waist circumference (men, B: -4.0 cm, p = 0.04; women, B: -3.4 cm, p = 0.06), compared to non-members.

**Conclusions:**

Health club membership is associated with significantly increased aerobic and resistance physical activity levels and more favorable cardiovascular health outcomes compared to non-members. However, longitudinal, randomized controlled trials would be clearly warranted as cross-sectional data prohibits causal inferences.

## Introduction

The United States Department of Health and Human Services has established specific goals to increase the number of individuals who meet the current physical activity (PA) guidelines [1]. Current evidence-based physical activity guidelines (PAG) recommend at least 150 minutes of moderate-intensity or 75 minutes of vigorous-intensity aerobic activity per week, or an equivalent combination of both [2]. The PAG also recommend participation in muscle-strengthening activities on 2 or more days per week for additional health benefits not found with aerobic activity [2]. However, most studies have focused on aerobic activity and more data are needed regarding the distribution and determinants of muscle-strengthening activities such as resistance exercise [3].

Despite the clear evidence of health benefits, approximately only half of Americans reported meeting the aerobic PAG, and only 30% reported meeting the muscle-strengthening PAG in 2013 [4]. The prevalence of meeting aerobic PAG is even lower (<10%) when assessed with objective measures of accelerometry [5]. These findings document the need for more effective population based strategies for promoting PA. Many worksite wellness programs in developed countries offer activity related programming with on-site exercise facilities [6]. Insurance coverage for a health club membership has also become more common in the US, and many companies even cover or subsidize the cost of health club memberships for their employees [6]. In addition, there are numerous commercial programs and health club facilities positioned to provide opportunities for PA promotion [7]. However, little is actually known about the impact of these facilities on health as most previous research has focused on other determinants of physical activity and sport [8–10]. One previous cross-sectional study has also simply compared the exercise levels of individuals with and without a health club membership [11], but no current literature has explored cardiovascular health in relation to health club membership. Further, there is very limited data about the associations of health club membership status and duration with the prevalence of meeting the current PAG (aerobic and/or muscle-strengthening PAG) and sedentary time.

A specific and practical question that needs to be addressed is whether, and to what extent, having a health club membership is associated with increased PA, higher fitness, and favorable cardiovascular health. In addition, there is little evidence about whether having a health club membership is associated with lifestyle PA outside health clubs or whether the duration of a health club membership is associated with PA levels, fitness, and health. Therefore, the purpose of this study was to determine the strength (magnitude) of the associations of the status and duration of a health club membership with the likelihood of meeting the aerobic and/or muscle-strengthening PAG, lifestyle PA levels, sedentary time, and cardiovascular health outcomes.

## Methods

### Study Participants

This study included 424 participants in which 19 were excluded due to incomplete data. After exclusions, 405 participants aged 30–64 years, who are expected to gain the most cardiovascular benefits from PA, remained for analyses. All participants were employed or residing in a college town (Ames, IA) with a population of approximately 63,000 [12]. The sample consisted of volunteer participants, who were predominantly white (85%), well-educated college faculty and staff members. Recruitment occurred using various methods, including flyers, e-mail, word of mouth, and in-person at the local college and 4 different commercial health clubs. Most health club members were recruited using the college faculty and staff email list and local health clubs, and non-members were recruited from the college faculty and staff without a health club membership. Participants were not on blood pressure medication (e.g., beta blockers) to minimize its effect on blood pressure and resting heart rate (main cardiovascular outcomes); did not have a history of heart attack, stroke, or cancer; and were not currently pregnant. We also collected personal history of physician-diagnosed diabetes, arthritis (rheumatoid or osteoarthritis), hypercholesterolemia, asthma, chronic obstructive pulmonary disease (COPD), and depression. We adjusted for these common medical conditions that may affect PA and/or cardiovascular disease (CVD) outcomes in our analyses. However, we did not exclude participants with these common, but less serious, medical conditions to increase generalizability of our study. Participants completed an evaluation including resting blood pressure, resting heart rate, waist circumference, weight and height measurement, as well as a PA and medical history questionnaire. Health club members were defined as those who reported that they had at least 1 month of health/fitness club membership at the time of study participation. Non-members were defined as those who reported they had no health/fitness club membership over the last 3 months. Iowa State University Institutional Review Board approved the study and all participants gave written informed consent.

### Cardiovascular Health Outcomes

Resting blood pressure and heart rate were measured using an Omron automated digital monitor (Omron BP760, Omron Healthcare, Inc., Lake Forest, IL) after at least a 10-minute seated rest period, with measurements on the upper left, bared arm. A minimum of two measurements were taken at intervals of at least 2 minutes, with additional measurements taken if a >5 mmHg difference occurred between the first and second reading in either systolic and/or diastolic blood pressure. Hypertension was defined as systolic or diastolic blood pressure ≥140/90 mmHg [13]. Increased resting heart rate was defined as a heart rate ≥80 b/min based on studies indicating increased risks of cardiovascular and all-cause mortality [14,15]. Participants recruited in the local health clubs completed resting blood pressure and heart rate measures before their exercise in the health club.

Body mass index (BMI) was determined from measured weight and height (kg/m^2^) using a standard stadiometer, and classified into 4 groups: underweight (<18.5 kg/m^2^), normal weight (18.5–24.9 kg/m^2^), overweight (25–29.9 kg/m^2^), or obese (≥30 kg/m^2^) [16]. Waist circumference (cm) was measured at the level of the umbilicus after exhalation. Abdominal obesity was defined as a waist circumference of >102 cm for men and >88 cm for women [17].

Cardiorespiratory fitness (maximal oxygen consumption in mL/kg/min) was estimated with a non-exercise algorithm incorporating age, BMI, waist circumference, resting heart rate, PA, and smoking status, based on a large longitudinal study in over 11,000 middle-aged adults [18]. This algorithm used to estimate a non-exercise testing cardiorespiratory fitness provides a valid indication of CVD and disease-specific mortality [19]. Low fitness was defined as <10 and <8 METs for 30–39 year old men and women, <9 and <7 METs for 40–49 years, <8 and <6 METs for 50–59 years, and <7 and <5 METs for ≥60 years, respectively, based on earlier studies indicating higher risks of CVD and mortality in people with the corresponding fitness levels [20,21]. Demographic variables, smoking status, alcohol consumption, dieting for weight loss, and personal history of common chronic diseases were obtained from the medical history question.

### Physical Activity Measurement

Self-reported PA was assessed using a questionnaire developed based on the International Physical Activity Questionnaire (IPAQ) [22]. Using the basic IPAQ format, separate questions were developed to capture both the frequency and duration of moderate aerobic PA, vigorous aerobic PA, and muscle-strengthening PA. Because the goal was to capture the association between health club membership and activity patterns, distinctions were made about whether the activity was performed in a health club setting or as part of their lifestyle using two separate PA sections: 1) PA performed only in the health club and 2) lifestyle PA performed outside the health club. Participants with a health club membership answered both health club and lifestyle PA questions (including occupation, housework, transportation, and leisure-time activities) over the past 30 days. Participants without a health club membership answered only lifestyle PA questions. Examples of health club aerobic activities included treadmill running or cycling, and muscle-strengthening activities included weight lifting using free weights, weight machines, or body weight (e.g., chin-ups or sit-ups). Examples of lifestyle aerobic activities included outdoor running, sport activities, or house cleaning, and muscle-strengthening activities included push-ups, sit-ups, or carrying heavy loads. Additionally, two questions asked about total sitting time on a typical week day and weekend day for all participants. Participants who spent ≥6 hours sitting per day, on average, were defined as sedentary, which has been identified as a CVD risk factor [23,24].

The items were scored, as follows, to compute overall activity levels and to determine compliance with the PAG. To calculate the total minutes of aerobic activities, the frequency was multiplied by the duration for each moderate and vigorous intensity activity, and summed over all activities. The duration of vigorous aerobic activity was multiplied by two based on the PAG, indicating that one minute of vigorous activity equals two minutes of moderate activity [2]. Muscle-strengthening activity was calculated as sessions per week. Participants were classified as meeting or not meeting the aerobic and/or muscle-strengthening PAG based on their total minutes of aerobic activities (≥150 minutes/week) and frequency (≥2 days/week) of muscle-strengthening activities. The adaptations and scoring of the items are consistent with IPAQ applications. There were significant Spearman correlation coefficients between total minutes of PA from our questionnaire and estimated cardiorespiratory fitness (*r* = 0.43, p<0.001) and resting heart rate (*r* = -0.32, p<0.001). Significant relationships between PA levels and these variables are generally expected, thus provide some potential support for validity of our PA questionnaire.

### Statistical Analyses

This was a cross-sectional study conducted between April and August 2013. All statistical analyses were conducted using SAS software (version 9.3, SAS Institute Inc; Cary, NC). Descriptive statistics for the two groups were compared using χ^2^ tests or *t* tests. Multivariable logistic and linear regression analyses were used to evaluate the associations of health club membership with PA and cardiovascular health outcomes, and are reported as odds ratio or beta coefficients (95% confidence intervals). Cardiovascular health outcome analyses were adjusted for age, sex, dieting for weight loss, BMI, disease presence (COPD, asthma, arthritis, diabetes, hypercholesterolemia, or depression), smoking status (current or non-smoker), and heavy alcohol consumption (>14 drinks/week for men, >7 drinks/week for women) [25]. All statistical tests were two-sided, and p<0.05 was accepted to indicate statistical significance.

## Results

There were 204 participants with a health club membership and 201 participants without a health club membership. Those with a health club membership were significantly more active and less sedentary (Table 1). Regarding total PA, including both health club and lifestyle activities, 87% and 84% of members compared to 30% and 36% of non-members met the aerobic PAG and resistance PAG, respectively. The prevalence of meeting both aerobic and resistance PAG was 75% in members and 18% in non-members in additional analysis. Those with a health club membership were also more fit and had a lower resting heart rate (Table 2).

**Table 1:** Demographic characteristics and physical activity by health club membership status.

	All	Member	Non-Member	P value
n	405	204	201	
Age (y)	47 (10)	47 (10)	47 (11)	0.99
Female sex (%)	227 (56)	113 (55)	91 (51)	0.84
Total Physical Activity (Lifestyle plus Health Club)				
Aerobic (min/week)	312 (368)	484 (397)	137 (227)	<0.001
0 (%)	66 (16)	4 (2)	62 (31)	<0.001
1–149 (%)	101 (25)	22 (11)	79 (39)	
150–299 (%)	87 (21)	55 (27)	32 (16)	
≥ 300 (%)	151 (37)	123 (60)	28 (14)	
Resistance (times/week)	2.8 (3.6)	3.9 (2.9)	1.6 (3.8)	<0.001
0 (%)	126 (31)	22 (11)	104 (52)	<0.001
1 (%)	36 (9)	11 (5)	25 (12)	
2 (%)	57 (14)	31 (15)	26 (13)	
≥3 (%)	186 (46)	140 (69)	46 (23)	
Lifestyle Physical Activity Only out of Health Club				
Aerobic (min/week)	148 (235)	160 (243)	137 (227)	0.33
0 (%)	128 (32)	66 (32)	62 (31)	0.67
1–149 (%)	150 (37)	71 (35)	79 (39)	
150–299 (%)	63 (16)	31 (15)	32 (16)	
≥ 300 (%)	64 (16)	36 (18)	28 (14)	
Resistance (times/week)	1.6 (3.1)	1.7 (2.2)	1.6 (3.1)	0.73
0 (%)	193 (48)	89 (44)	104 (52)	0.48
1 (%)	49 (12)	24 (12)	25(12)	
2 (%)	58 (14)	32 (16)	26 (13)	
≥3 (%)	105 (26)	59 (28)	46 (23)	
Total Membership Period (years)	-	6.3 (7.8)	0	
<2 (%)	77 (19)	77 (38)	-	
2–6 (%)	64 (16)	64 (31)	-	
>6 (%)	63 (15)	63 (31)	-	
Sitting Time (hours/day)	6.9 (2.9)	6.2 (2.7)	7.7 (2.9)	<0.001
Sedentary (%)^a^	272 (67)	119 (58)	153 (76)	<0.001
Current Smoker (%)	7 (2)	4 (2)	3 (1)	1
Diet to Lose Weight (%)	43 (11)	24 (12)	19 (9)	0.52
Disease Presence (%)^b^	122 (30)	60 (30)	62 (31)	0.75
Heavy Alcohol Drinking (%)^c^	11 (3)	6 (3)	5 (2)	1

Data are presented as mean (SD) for continuous variables and n (%) for categorical variables

^a^ Defined as sitting ≥6 hours per day

^b^ Defined as having previous diagnosis of chronic obstructive pulmonary disease, asthma, arthritis (rheumatoid or osteoarthritis), diabetes, hypercholesterolemia, or depression

^c^ Defined as >7 drinks/week for women and >14 drinks/week for men as defined by the National Institute on Alcohol Abuse and Alcoholism [25]

**Table 2:** Cardiovascular health outcomes by health club membership status.

	All	Member	Non-Member	P value
Body Mass Index (kg/m^2^)	27.0 (5.3)	26.9 (5.3)	27.1 (5.3)	0.59
<18.5 (%)	5 (1)	2 (1)	3 (1)	0.15
18.5–24.9 (%)	168 (41)	86 (42)	82 (41)	
25–29.9 (%)	133 (33)	75 (37)	58 (29)	
≥30 (%)	99 (24)	41 (20)	58 (29)	
Waist Circumference (cm)				
Male	96.0 (12.2)	94.8 (11.7)	97.2 (12.7)	0.20
Female	87.6 (13.4)	86.5 (13.5)	88.7 (13.2)	0.21
Abdominal obesity (%)^a^				
Male	53 (30)	25 (14)	28 (16)	0.49
Female	95 (42)	45 (20)	50 (22)	0.54
Resting Heart Rate (b/min)	69 (11)	67 (10)	71 (10)	<0.001
Increased Resting Heart Rate (%)^b^	66 (16)	24 (12)	42 (21)	0.01
Systolic Blood Pressure (mmHg)	115 (16)	115 (14)	116 (17)	0.49
Diastolic Blood Pressure (mmHg)	76 (10)	75 (9)	77 (11)	0.20
Hypertension (%)^c^	46 (11)	19 (9)	27 (13)	0.19
Cardiorespiratory Fitness (mL of O_2_/kg/min)	36.4 (7.2)	37.6 (10.8)	35.1 (7.3)	<0.001
Unfit (%)^d^	10 (3)	4 (2)	6 (3)	0.51

Data are presented as mean (SD) for continuous variables and n (%) for categorical variables.

^a^ Defined as waist circumference >88 cm in women, >102 cm in men

^b^ Defined as a resting heart rate ≥80 b/min

^c^ Defined as systolic/diastolic blood pressure ≥140/90 mmHg

^d^ Defined as <10 and <8 METs for 30–39 year old participants, <9 and <7 METs 40–49 years, <8 and <6 METs for 50–59 years, and <7 and <5 METs for ≥60 year men and women, respectively

**Fig 1:**
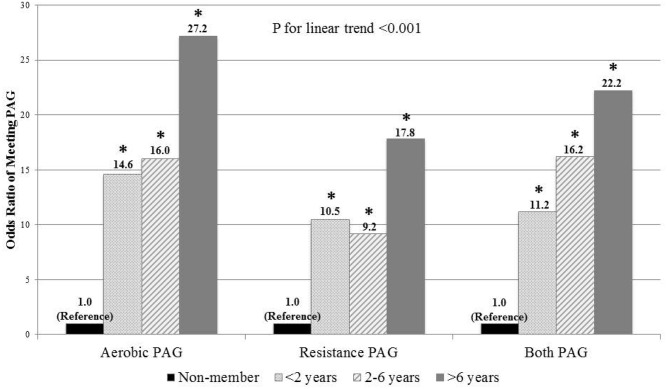
Odds ratios of meeting physical activity guidelines (PAG) by length of health club membership using total physical activity. The reference category for all analyses was non-health club members. All analyses were adjusted for age, sex, and body mass index. * indicates significantly different from the reference category, p<0.05. Odds ratios (95% confidence intervals) were 14.6 (6.9–30.9), 16.0 (7.2–35.7), and 27.2 (10.3–72.0) in meeting the aerobic PAG, 10.5 (5.1–21.4), 9.2 (4.5–18.7), and 17.8 (7.2–43.9) in meeting the resistance PAG, and 11.2 (5.9–21.3), 16.2 (8.1–32.5), and 22.2 (10.4–47.3) in meeting both aerobic and resistance PAG.

In logistic regression analyses (Table 3), the odds ratios (OR) (95% confidence interval [CI]) of meeting PAG in health club members compared to non-members were 16.5 (9.8–27.6) for aerobic, 10.1 (6.2–16.3) for resistance, and 13.8 (8.5–22.4) for both PAG using total PA (health club PA plus lifestyle PA) after adjusting for age, sex, and BMI. However, there were no significant differences in the odds of meeting the PAG when comparing only lifestyle PA outside the health club between members and non-members (p>0.05). This result suggests that health club members have similar, but not lower, PA levels outside their health clubs compared to non-members. A significant linear trend (p<0.001) was observed for increasing duration of health club membership and meeting the PAG (Fig 1).

**Table 3:** Health club membership and meeting the physical activity guidelines.

	Met Aerobic Guidelines	Met Resistance Guidelines	Met Both Guidelines
**Using Total Physical Activity**			
Unadjusted Odds Ratio (95% CI)			
Non-Health Club Member	1.0 (reference)	1.0 (reference)	1.0 (reference)
Health Club Member (All)	16.1 (9.7–26.8)	9.7 (6.0–15.6)	13.3 (8.3–21.4)
Male^b^	13.8 (6.5–29.5)	10.3 (4.9–12.6)	11.2 (5.6–22.6)
Female^c^	18.1 (0.1–36.2)	9.5 (5.1–17.6)	15.4 (8.0–29.8)
Adjusted Odds Ratio (95% CI)^a^			
Non-Health Club Member	1.0 (reference)	1.0 (reference)	1.0 (reference)
Health Club Member (All)	16.5 (9.8–27.6)	10.1 (6.2–16.3)	13.8 (8.5–22.4)
Male^b^	14.1 (6.6–30.3)	10.5 (4.9–22.3)	11.6 (5.7–23.5)
Female^c^	18.4 (9.1–37.1)	9.7 (5.2–18.2)	15.8 (8.1–30.9)
**Using Lifestyle Physical Activity Only**			
Unadjusted Odds Ratio (95% CI)			
Non-Health Club Member	1.0 (reference)	1.0 (reference)	1.0 (reference)
Health Club Member (All)	1.1 (0.8–1.8)	1.4 (1.0–2.2)	1.1 (0.7–1.9)
Male^b^	1.1 (0.6–2.0)	1.5 (0.8–2.6)	1.0 (0.5–2.1)
Female^c^	1.2 (0.7–2.1)	1.4 (0.8–2.5)	1.3 (0.6–2.6)
Adjusted Odds Ratio (95% CI)^a^			
Non-Health Club Member	1.0 (reference)	1.0 (reference)	1.0 (reference)
Health Club Member (All)	1.1 (0.7–1.7)	1.4 (0.9–2.1)	1.1 (0.7–1.9)
Male^b^	1.1 (0.6–2.0)	1.4 (0.8–2.6)	1.0 (0.5–2.0)
Female^c^	1.2 (0.6–2.1)	1.4 (0.8–2.4)	1.2 (0.6–2.5)

CI: Confidence Interval

^a^ Adjusted for age, sex, and body mass index

^b^ Reference group was male non-health club members.

^c^ Reference group was female non-health club members.

Table 4 shows the odds ratio of cardiovascular health outcomes in health club members compared to non-members. Health club membership was associated with significantly lower odds (OR, 95% CI) of elevated resting heart rate (0.5, 0.3–0.9) and obesity (0.6, 0.4–0.97) after adjusting for potential confounders. Although not significant, individuals with a health club membership also had lower odds of hypertension (0.7, 0.3–1.4) and abdominal obesity (men: 0.7, 0.3–1.4; women: 0.8, 0.5–1.5). Due to the small number of unfit participants (n = 4 in members and n = 6 in non-members), cardiorespiratory fitness was not included in this analysis. After further adjustment for total PA in additional analyses, the associations for all outcomes were no longer significant, indicating the potential cardiovascular benefits of having a health club membership are most possibly from increased PA, especially at the health club (p>0.05). We further analyzed the data by removing individuals with a health club membership for 1 year or less, under the assumption that they may not yet fully display the benefits of PA. This analysis resulted in more robust relationships with lower odds (OR, 95% CI) of an elevated resting heart rate (0.4, 0.2–0.8), obesity (0.5, 0.3–0.9), and abdominal obesity (men: 0.5, 0.2–1.1; women: 0.7, 0.4–1.3).

**Table 4:** Odds ratios of cardiovascular health outcomes by health club membership status in all members and members with over 1 year of membership.

	Adjusted Odds Ratio (95% Confidence Interval)^a^		
	Non-Members	All Members	Members >1 year
Increased Resting Heart Rate^b^	1.0 (Reference)	0.5 (0.3–0.9)	0.4 (0.2–0.8)
Hypertension^c^	1.0 (Reference)	0.7 (0.3–1.4)	0.9 (0.4–1.8)
Obesity^d^	1.0 (Reference)	0.6 (0.4–0.97)	0.5 (0.3–0.9)
Abdominal Obesity^e^			
Men	1.0 (Reference)	0.7 (0.3–1.4)	0.5 (0.2–1.1)
Women	1.0 (Reference)	0.8 (0.5–1.5)	0.7 (0.4–1.3)

In all logistic regression analyses, non-members were the reference group.

^a^ Adjusted for age, sex, dieting for weight loss, body mass index (not for obesity or abdominal obesity), smoking status, disease presence (yes or no), and heavy alcohol consumption.

^b^ Defined as resting heart rate of ≥80 b/min

^c^ Defined as systolic/diastolic blood pressure of ≥140/90 mmHg

^d^ Defined as body mass index of ≥30 kg/m^2^

^e^ Defined as waist circumference >102 cm for men and >88 cm for women.

In multivariable linear regression analysis, significant health benefits were found for individuals with a health club membership on resting heart rate, cardiorespiratory fitness, and waist circumference (Table 5). Individuals with a health club membership had a 4.8 b/min lower resting heart rate (B: -4.8, p<0.001) and 2.1 mL/kg/min higher cardiorespiratory fitness (B: 2.1, p<0.001), after adjusting for the potential confounders. Removal of individuals with a health club membership of 1 year or less resulted in more favorable findings in most outcomes, especially waist circumference (men, B: -4.0 cm, p = 0.04; women, B: -3.4 cm, p = 0.06). However, statistical significance was lost after further adjustment for total PA in all outcomes (p>0.05). Although not significant, individuals with a health club membership had lower systolic and diastolic blood pressure and lower BMI. In regards to PA, members spent approximately 5.8 hours/week (B: 5.8, p<0.001) being more aerobically active, 2.3 times/week (B: 2.3, p<0.001) doing more muscle-strengthening PA, and 1.4 hours/day (B: -1.4, p<0.001) being less sedentary, compared to non-members.

**Table 5:** Differences in cardiovascular health and physical activity by health club membership.

Outcome	Health Club Membership		Health Club Membership >1 year^a^	
	B (95% CI)	p-value	B (95% CI)	p-value
Cardiovascular Health^b^				
Resting Heart Rate (b/min)	-4.8 (-6.8, -2.8)	<0.001	-5.3 (-7.4, -3.2)	<0.001
Cardiorespiratory Fitness (mL of O_2_/kg/min)	2.1 (1.6, 2.5)	<0.001	2.3 (1.8, 2.8)	<0.001
Systolic Blood Pressure (mmHg)	-0.7 (-3.2, 1.7)	0.56	-0.4 (-3.1, 2.3)	0.79
Diastolic Blood Pressure (mmHg)	-1.0 (-2.7, 0.6)	0.23	-0.5 (-2.3, 1.3)	0.60
Body Mass Index (kg/m^2^)	-0.3 (-1.3, 0.7)	0.50	-0.8 (-1.9, 0.3)	0.16
Waist Circumference (cm)				
Male	-3.1 (-6.5, 0.3)	0.07	-4.0 (-7.8, -0.2)	0.04
Female	-2.1 (-5.4, 1.3)	0.22	-3.4 (-6.9, 0.2)	0.06
Physical Activity^c^				
Aerobic Activity (hours/week)	5.8 (4.7, 6.8)	<0.001	6.6 (5.4, 7.8)	<0.001
Resistance Activity (times/week)	2.3 (1.7, 3.0)	<0.001	2.3 (1.6, 3.1)	<0.001
Sedentary Time (hours/week)	-1.4 (-1.9, -0.8)	<0.001	-1.3 (-1.9, -0.7)	<0.001

CI: confidence interval;

In all multivariable linear regressions, we used non-members as reference group.

^a^ In this subsample analyses, we included health club members with over one year of membership.

^b^ Adjusted for age, sex, body mass index (not for body mass index or waist circumference), smoking status, disease presence (yes or no), dieting for weight loss, and heavy alcohol consumption.

^c^ Adjusted for age, sex, and body mass index.

## Discussion

The primary finding of this study is the significant associations of health club membership with increased aerobic and resistance PA levels as well as favorable cardiovascular health outcomes including resting heart rate, cardiorespiratory fitness, and obesity measures. Additionally, an increased length of membership was more strongly associated with increased physical activity and favorable cardiovascular health outcomes.

Our findings are consistent with a previous cross-sectional study comparing fitness center members and non-members in which fitness center members exercised more regularly (88% of members compared to 54% of non-members), at higher intensities, and for longer periods of time (64 minutes vs. 54 minutes per session, p<0.001) [11]. In our study, the number of minutes of additional aerobic PA in the health club members (484 minutes/week) is drastically larger than those without memberships (137 minutes/week) (Table 1). The difference between the previous study and the current study may be related to differences in PA questionnaires (e.g., more comprehensive questionnaire in our study vs. a simple 5-question Health-Promoting Lifestyle Profile without information about muscle-strengthening activity and separate comparison of health club and lifestyle PA), population (e.g., higher education levels in our study), and study design (e.g., in-person data collection in our study vs. mailed questionnaires).

Our study also investigated the association of health club membership with resistance PA. In community-dwelling adults, meeting the resistance PAG is more challenging without access to a health club, where various weight training equipment is typically available. Our study suggests positive associations of a health club membership with meeting the resistance PAG, and both aerobic and resistance PAG. Only 36% and 18% of non-members met resistance PAG and both aerobic and resistance PAG, respectively, which greatly differs from the 84% and 75% of members meeting the respective guidelines. This results in 10 and 14 times higher odds of meeting resistance PAG and both PAG, respectively, in members compared to non-members (Table 3). However, due to the cross-sectional study design, it is also possible that active people may be more inclined to join a health club. Therefore, further prospective studies are needed to investigate a causal relationship between health club membership and PA.

It may be expected that health club members would have lower amounts of other PA performed at home, work, or during leisure-time outside their health club because they may feel satisfied with their health club activities. However, our results show that health club members had similar activity levels outside the health club compared to non-members. Thus, having a health club membership does not appear to be associated with reduced lifestyle PA levels, but only increased total PA.

In this study, favorable CVD health markers were observed in those with health club memberships, such as lower resting heart rate, lower odds of being obese, increased cardiorespiratory fitness, and reduced sedentary time compared to non-members. However, we found that these positive associations between health club memberships and favorable CVD markers were no longer significant after further adjustment for total PA, which possibly indicates the potential benefits of PA as a mediator between health club membership and favorable CVD markers. This finding is supported by the current PAG, indicating health benefits of PA, such as reduced risk of CVD, metabolic syndrome, high cholesterol, osteoporosis, sarcopenia, and functional impairment [20,26–28]. Our findings suggest that having a health club membership may be an effective way to increase PA for cardiovascular health benefits. Unfortunately, only 18.2% of Americans have a health club membership [29]. Ideally more people with active memberships would result in increased PA and more favorable health and fitness, as well as reduced sedentary time, which is an important and independent emerging risk factor for cardiovascular health and premature mortality [24,30,31]. Recent meta-analyses have suggested that increased sedentary time (e.g., sitting, watching TV) is associated with increased risks of type 2 diabetes, cardiovascular disease, hospitalization, and all-cause mortality regardless of PA levels [23,31–33].

In this study, more favorable cardiovascular health outcomes were generally noted in health club members, compared to non-members, after excluding health club members with ≤1 year of health club membership (Tables 4 and 5). These findings possibly suggest that retaining a health club membership is associated with accomplishing health goals, such as to lose weight or lower blood pressure, as individuals who may have recently purchased a health club membership may not have been physically active for an adequate duration to see the health benefits yet.

It is also possible that the PA performed at the health club is quite different from the lifestyle PA outside the health club, which could contribute to the positive outcomes, although specific activities were not collected in the current study. Many health clubs offer organized classes, such as yoga, spinning, Pilates, etc., which have gained popularity. For instance, in 2013, one in five health club members participated in yoga classes, followed in popularity by aerobics and group cycling [29]. It is possible that this group exercise approach may create a synergy about the class, bringing motivation and encouragement from other members, ultimately resulting in greater PA in health club members. However, future studies and data are clearly needed on this topic.

### Limitations

The results should be interpreted with caution as cross-sectional data prohibits causal inferences, thus it is also possible that more active individuals purchase health club memberships. However, to examine more long term prospective associations between health club membership and PA, fitness, and cardiovascular health, we included health club members with at least 1 month of membership, and investigated whether a longer duration of membership is associated with those outcome variables. The majority of study participants were white (85%), well-educated university faculty and staff, recruited from Ames, Iowa from April to August, and unfortunately markers of socioeconomic status such as annual income were not collected. Therefore, results may vary in other populations in different places during different seasons of the year, and may be affected by socioeconomic status. Additionally, the use of self-reported PA may introduce measurement error. However, people generally over report their PA, and this over-reporting underestimates the true health effects of PA. Therefore, future studies using an objective measure of PA in the health club would strengthen the associations between health club membership and cardiovascular health outcomes. In addition, further studies are needed to investigate the effects of the patterns of change in health club membership status on PA and cardiovascular health outcomes using multiple assessments for those variables over time. Another limitation is that we measured waist circumference at the umbilicus level instead of midway between the lowest ribs and the iliac crest, which is clinically more recommended. Therefore, the result on waist circumference should be carefully interpreted and applied.

## Conclusion

In conclusion, this study suggests that health club membership was associated with increased PA and more favorable CVD health markers, especially with longer duration of membership. With the declining average monthly fee for health club membership [29] and the increasing support from employers and insurance companies, this study supports that promoting PA at a health club could be an effective public health strategy for health promotion and potentially health care cost reduction. However, large longitudinal, randomized controlled trials are clearly warranted to confirm our findings.
